# Automated detection of spinal bone marrow oedema in axial spondyloarthritis: training and validation using two large phase 3 trial datasets

**DOI:** 10.1093/rheumatology/keaf323

**Published:** 2025-06-09

**Authors:** Amir Jamaludin, Rhydian Windsor, Sarim Ather, Timor Kadir, Andrew Zisserman, Juergen Braun, Lianne S Gensler, Mikkel Østergaard, Denis Poddubnyy, Thibaud Coroller, Brian Porter, Gregory Ligozio, Aimee Readie, Pedro M Machado

**Affiliations:** Visual Geometry Group, Department of Engineering Science, University of Oxford, Oxford, UK; Visual Geometry Group, Department of Engineering Science, University of Oxford, Oxford, UK; Oxford University Hospitals NHS Foundation Trust, Oxford, UK; Selka Health, Oxforrd, UK; Visual Geometry Group, Department of Engineering Science, University of Oxford, Oxford, UK; Rheuma Praxis Berlin, Berlin, Germany; Ruhr-Universität Bochum, Bochum, Germany; Division of Rheumatology, University of California San Francisco, San Francisco, CA, USA; Copenhagen Centre for Arthritis Research, Centre for Rheumatology and Spine Diseases, Rigshospitalet, Glostrup, Denmark; Department of Clinical Medicine, University of Copenhagen, Copenhagen, Denmark; Division of Rheumatology, Department of Medicine, University Health Network and University of Toronto, Toronto, Canada; Department of Gastroenterology, Infectiology and Rheumatology (including Nutrition Medicine), Charité – Universitätsmedizin Berlin, corporate member of Freie Universität Berlin and Humboldt-Universität zu Berlin, Berlin, Germany; Novartis Pharmaceuticals Corporation, East Hanover, NJ, USA; Novartis Pharmaceuticals Corporation, East Hanover, NJ, USA; Novartis Pharmaceuticals Corporation, East Hanover, NJ, USA; Novartis Pharmaceuticals Corporation, East Hanover, NJ, USA; Department of Neuromuscular Diseases, UCL Queen Square Institute of Neurology, University College London, London, UK; National Institute for Health Research (NIHR), University College London Hospitals Biomedical Research Centre, London, UK; Department of Rheumatology, Northwick Park Hospital, London North West University Healthcare NHS Trust, London, UK

**Keywords:** axial spondyloarthritis, ankylosing spondylitis, MRI, inflammation, bone marrow oedema, osteitis, machine learning, artificial intelligence

## Abstract

**Objective:**

To evaluate the performance of machine learning (ML) models for the automated scoring of spinal MRI bone marrow oedema (BMO) in patients with axial spondyloarthritis (axSpA) and compare them with expert scoring.

**Methods:**

ML algorithms using SpineNet software were trained and validated on 3483 spinal MRIs from 686 axSpA patients across two clinical trial datasets. The scoring pipeline involved (i) detection and labelling of vertebral bodies and (ii) classification of vertebral units for the presence or absence of BMO. Two models were tested: Model 1, without manual segmentation, and Model 2, incorporating an intermediate manual segmentation step. Model outputs were compared with those of human experts using kappa statistics, balanced accuracy, sensitivity, specificity and AUC.

**Results:**

Both models performed comparably to expert readers, regarding presence *vs* absence of BMO. Model 1 outperformed Model 2, with an AUC of 0.94 (*vs* 0.88), accuracy of 75.8% (*vs* 70.5%) and kappa of 0.50 (*vs* 0.31) using absolute reader consensus scoring as the external reference; this performance was similar to the expert inter-reader accuracy of 76.8% and kappa of 0.47 in a radiographic axSpA dataset. In a non-radiographic axSpA dataset, Model 1 achieved an AUC of 0.97 (*vs* 0.91 for Model 2), accuracy of 74.6% (*vs* 70%) and kappa of 0.52 (*vs* 0.27), comparable to the expert inter-reader accuracy of 74.2% and kappa of 0.46.

**Conclusion:**

ML software shows potential for automated MRI BMO assessment in axSpA, offering benefits such as improved consistency, reduced labour costs and minimized inter- and intra-reader variability.

**Trial registration:**

Clinicaltrials.gov, http://clinicaltrials.gov, MEASURE 1 study (NCT01358175); PREVENT study (NCT02696031).

Rheumatology key messages

- In axial spondyloarthritis (axSpA), machine learning (ML)-based bone marrow oedema (BMO) detection in whole-spine MRI matches expert performance.
- This ML system enhances consistency, improving accuracy, reducing labour costs and minimizing variability.
- This ML system could facilitate consistent MRI assessment in large-scale epidemiological studies of axSpA.

## Introduction

Magnetic resonance imaging (MRI) is a commonly used method for assessing and monitoring axial spondyloarthritis (axSpA) [1–5]. MRI offers a non-invasive and objective approach for early diagnosis and classification, as well as monitoring disease burden and treatment response in patients with axSpA [6, 7].

Quantification of MRI changes has become a valuable tool in demonstrating the efficacy of biologic and targeted-synthetic disease-modifying anti-rheumatic drugs (DMARDs) in the treatment of axSpA. Various scoring systems exist for semi-quantitative assessment of acute and chronic MRI changes in axSpA. However, they are all prone to subjectivity and, therefore, intra- and inter-reader variability; they require a level of expertise that is not widely available; and they are labour-intensive and costly [6].

Recently, machine learning (ML) algorithms have been explored as a potential solution to improve the accuracy and efficiency of MRI assessment. ML is a type of artificial intelligence (AI) that can automatically detect patterns in data [8]. For example, computer-aided risk stratification using ML classification of benign and malignant cancer nodules has been suggested as a potential method for aiding in lung cancer diagnosis [9].

ML efforts at the spinal level have focused on developing automated systems for detecting and quantifying MRI disc degeneration [10–13], with few studies addressing the sacroiliac joints (SIJ) in axSpA [14–21]. Only one recent study has addressed the automated detection of bone marrow oedema (BMO) in the spine of axSpA patients [22]. However, technical approaches have been diverse, and interpreting results from these automated systems can be challenging due to the lack of standardization and variability in the interpretation of ground-truth definitions [14–22].

In this study, we aimed to evaluate the performance of a ML-based software for automated detection of spinal MRI BMO in axSpA patients and compare this with expert assessment.

## Methods

### Oedema detection pipeline

There are two main steps in the proposed BMO detection pipeline for whole spine MRIs:

- The first step involves detecting and labelling the vertebral bodies in sagittal MRI images of the whole spine. Detection includes identifying the coordinates of each corner of the vertebral bodies, while labelling assigns a label to each detection; details of this pipeline are discussed in [23]. Vertebral units (VUs), defined as the area between the midpoints of two adjacent vertebral bodies, are then extracted from these detections using the detected and labelled vertebral bodies.
- The second step involves classifying the extracted VUs. Each VU contains halves of two vertebrae, and the classification process determines whether oedema is present in each VU.

### Datasets used for the oedema detection pipeline

The primary datasets used to develop the vertebral body detection and labelling pipeline (first step) were Genodisc and Oxford Whole Spine. The two main studies used to build and validate the oedema detection process (second step) were MEASURE 1 [24] and PREVENT [25].

1. Genodisc: This dataset is a large collection of lumbar MRI scans, comprising 2287 MRI assessments from 2009 patients, with sagittal slices from multiple clinical centres across Europe. Each vertebra from S1 to T12 has been annotated as a series of polygons across sagittal slices, and each scan has been labelled for various spinal degenerative changes by an expert radiologist. The Genodisc dataset is divided into training, validation and testing sets, based on the number of patients, with 1880, 203 and 204 MRI assessments in each set, respectively.
2. Oxford Whole Spine (OWS): The dataset consists of 710 anonymized whole-spine scans from 196 patients, collected from the PACS digital imaging platform of the Oxford University Hospitals NHS Foundation Trust. These scans include T1, T2 and STIR sequences, with each vertebral body from c2 to S1 annotated by a non-specialist in the central slice of each scan. The OWS dataset is divided into training, validation and testing sets in an 80:10:10 ratio, based on the number of patients rather than the number of MRI assessments.
3. MEASURE 1: Details of the phase III MEASURE 1 study (NCT01358175) have previously been published [24]. Further MEASURE 1 patient population details are presented in Supplementary Data S1 (available at *Rheumatology* online). We included 131 patients from the MEASURE 1 study [24], in which T1 and STIR sagittal spinal MRIs were taken at various time points (baseline, weeks 16, 52, 104, 156, 208 and some unscheduled scans). Two expert readers, R1 and R2 (both with >10 years clinical and research experience scoring MRI of axSpA clinical trials) evaluated all the images blinded to treatment assignment and image sequence, and assigned BMO scores according to the Berlin modification of the Ankylosing Spondylitis Spine Magnetic Resonance Imaging activity (ASspiMRIa) scoring system; in the Berlin scoring system, individual oedema lesions are scored on a scale of 0 to 3 for every VU assessed. For this work, these scores were binned into two categories: oedema absent (Berlin score 0) versus present (Berlin score of either 1, 2 or 3, representing presence of oedema to an increasing extent). The complete dataset included 131 patients and 562 MRI scans, totalling 12 877 VUs, most of which were graded by both R1 and R2. There were three separate read sessions for each reader: the first session (session 1) covered scans from Baseline to Week 52, the second session (session 2) covered scans from Baseline to Week 104, and the final session (session 3) covered all the scans in the dataset. The MRI spine protocol is presented in Supplementary Data S1 (available at *Rheumatology* online).
4. PREVENT: Details of the phase III PREVENT study (NCT02696031) have previously been published [25]. Further PREVENT patient population details are presented in Supplementary Data S1 (available at *Rheumatology* online). We included 555 patients from the PREVENT study, with 2921 MRI scans, totalling 66 821 VUs. The imaging protocol, reader experience level and reading rules were similar to that used in MEASURE 1, and this dataset also includes multiple time points (baseline, weeks 16, 52, 104 and some unscheduled scans). This dataset was used as a completely held-out dataset to validate the models developed on MEASURE 1. Similar to MEASURE 1, PREVENT also includes two readers and three read sessions. The MRI spine protocol is presented in Supplementary Data S1 (available at *Rheumatology* online).

### First step of the pipeline: detection and labelling

The first step in our pipeline involves detecting and labelling 23 vertebrae, specifically the vertebral bodies (VBs). From two pairs of vertebrae, we define and use a vertebral unit (VU) made from the lower and upper halves of the vertebrae. The algorithm is based on a previously developed software called SpineNet [13] and its updated variant, SpineNetV2 [13, 26]. This part of the pipeline is discussed in more detail by Windsor *et al.* [23] but can be summarized into two main parts: (i) detection and (ii) labelling. An overview of this method is shown in Supplementary Figs S1 and S2 (available at *Rheumatology* online).

#### Detection

To detect vertebral bodies in raw scans, we used a neural network model that predicts four corner points of the vertebral bodies, which represents the vertebrae as quadrilaterals. This is done across the sagittal slices (both in T1 and STIR sequences) where the vertebra is visible. The model was trained with vertebral bodies with variations in size and shape. At the output stage, these quadrilaterals, which are detected in each sagittal slices, are grouped across slices based on their intersection-over-union (IOU) to form three-dimensional (3D) volumes for each visible VB, starting from the slice with the most VB detections and expanding outwards to construct 3D volumes for all visible vertebrae.

#### Labelling

This step is crucial for determining the vertebral levels of the detected vertebral bodies. The task is challenging because scans may not be constrained to a single field of view, making traditional ‘counting up’ methods that rely on visible anchor vertebrae ineffective. To label the vertebrae, both appearance (e.g. intensity, shape, size) and context (position relative to other detections) are considered. Appearance information generates a probability vector for each detection, which is used to construct a probability-height map. This map is refined using a convolutional context network that incorporates the appearance predictions of nearby vertebrae. The resulting probability-height map is decoded into discrete-level predictions using a beam search that evaluates the probability score of each sequence and penalizes sequences with transitional vertebrae or numerical variations. This process enables the labelling of each visible vertebra in the scan, facilitating tasks such as measuring spinal curvature or extracting regions of interest for downstream classification.

From two consecutive VBs, we extract a VU region that includes both halves of the VB pair. This approach is used because axSpA BMO commonly occurs on the upper and lower endplates of the vertebrae, and it aligns with how the corresponding Berlin scores for each VU were read, as described above.

### Second step of the pipeline: bone marrow oedema detection

**Figure 1.**
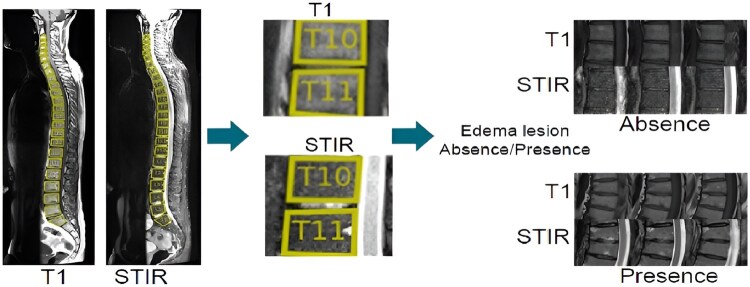
Overall bone marrow oedema automated detection process. In the examples on the right, three slices of T1 and STIR images are displayed; however, our models process all the slices for a given volume unit, ensuring a thorough analysis and accurate representation of the entire anatomical structure

From each detected and labelled VU, a binary representation of the Berlin modification of the ASspiMRIa score is created by the model, with each VU scored as either 0 (oedema absent) or 1 (oedema present, derived from the original Berlin scores of 1, 2 and 3). To accurately classify each VU, both T1 and STIR sequences are required. Fig. 1 provides an overview of the BMO automated detection process.

We tested two model training approaches.

**Figure 2.**
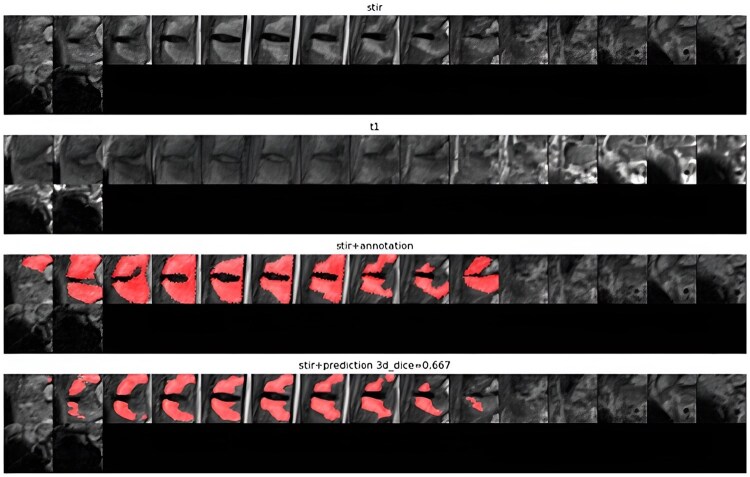
Quantitative examples of MEASURE 1, including both the annotation by the expert radiologist and the model annotation prediction. First panel, STIR sequence; second panel, T1-weighted sequence; third panel, annotation by the expert in the STIR sequence; fourth panel, model annotation prediction in the STIR sequence

1. Model 1 (consensus-based training): Training the model using individual readers from separate sessions might not be optimal and could result in increased noise, so we utilized a noise-reduction approach (closer to the ‘ground truth’) where the model learned from ‘consensus’ readings.
  Consensus labels were determined for each reader (R1 and R2) based on agreement across three reading sessions per VU:
  - if a VU was consistently scored as positive (1, 2, or 3) across all three sessions, it was marked as positive;
  - if all three sessions consistently scored it as negative, it was marked as negative; and
  - if there was no agreement across all three read sessions, the VU was labelled inconclusive and excluded from training and testing.
  Additionally, we introduced ‘super-consensus’ grading, where VUs consistently identified as having BMO across sessions by both R1 and R2 were used for training.
  The ML-classified VUs were compared against the human-annotated consensus assessments for each reader. This allowed us to evaluate the performance of the ML system against the consensus scores of individual readers. A ResNet-based [28] model with multiple classification heads, one each for each reader and the corresponding read session, was used.
2. Model 2 (segmentation-based training): We also explored directly segmenting BMO on the scans themselves. An expert reader (adjudicator reader, different from the original MEASURE 1 readers) was tasked with delineating regions of interest (ROIs) in positive Berlin cases using the scores from the two readers over the three read sessions as guidance. We trained a modified U-Net network [28], which takes the same 3D input as the Berlin classification models and outputs segmentation masks delineating the BMO regions for each VU. The subset of data reannotated with lesion segmentation masks comprised 1208 VUs, fully annotated slice-by-slice from 95 patients. Fig. 2 shows quantitative examples from MEASURE 1, including both the annotation by the expert radiologist and the model annotation prediction.

### Statistical analysis

Data from MEASURE 1 were used for training, validation and testing, while PREVENT data were kept as a testing-only dataset.

To train the detection models, we used 10-way cross-validation. The dataset was split into 10 folds of data; the ML model was trained on eight folds, validated on a single separate fold and tested on the remaining fold. This process was repeated 10 times, with different rotations of the 10 folds each time. The results were then calculated using the separate 10 test-set folds. This method allows for a more robust evaluation of the model’s performance by ensuring that all the data are used for both training and testing. For the reannotated segmentation experiment, a five-way cross-validation was used.

Model performance was assessed by comparing the results of the ML models (Models 1 and 2) with human reading results (i.e. ML model binary BMO assessment *vs* R1, R2, consensus and super-consensus binary BMO assessment) and using human performance (intra-reader: binary BMO assessment of R1 *vs* R1 and of R2 *vs* R2; and inter-reader: binary BMO assessment of R1 *vs* R2) as a benchmark for interpretation. In the ML model *vs* human reading comparison, kappa statistics, balanced accuracy, sensitivity, specificity and AUC were calculated using the human readers’ scores as the gold standard. In the expert intra- and inter-reader comparison, kappa statistics, balanced accuracy, sensitivity and specificity were calculated using one of the sessions (intra-reader) or one of the readers (inter-reader) as the external reference.

Kappa provides a measure of agreement beyond chance, with values ranging from −1 to 1, where 0 indicates agreement equivalent to chance and higher positive values indicate stronger agreement. Balanced accuracy is the average per-class accuracy, which is equal to (Sensitivity + Specificity)/2. Specificity, or the true negative rate, refers to the percentage of negatives correctly identified as such by the system compared with human reading, or one reader compared with the other. Sensitivity, or the true positive rate, refers to the percentage of positives correctly identified as such by the system compared with human reading, or one reader compared with the other. The AUC provides a summary measure of overall accuracy, with values ranging from 0.5 (no discrimination) to 1.0 (perfect discrimination). Of note, the readers designated as R1 and R2 in MEASURE 1 and PREVENT were not the same individuals, and both Models 1 and 2 were trained on MEASURE 1 data only, with the same models developed in MEASURE 1 applied to the PREVENT dataset without any additional training or fine-tuning.

### Ethics approval

This study uses clinical trial data. The trial protocols were approved by ethics review boards at each study site. Trials were performed in accordance with the ethical principles of the Declaration of Helsinki. Because both the clinical trial data and the images are anonymized, these are no longer personal data and so no further EC/IRB (Ethics Committees/Institutional Review Board) approvals are required. Neither patients nor the public were involved in the design of this study.

## Results

### Detection and labelling

The main results on the detection and labelling pipeline have been previously published [26]; the key takeaways are that the detection stage has a precision or positive predictive value of 99.0–99.7% and a recall or sensitivity of 98.1–99.1%, while the labelling stage has an accuracy of 90.9–98.4% (dataset-dependent). No further fine-tuning was needed to apply the detection and labelling pipeline to MEASURE 1 and PREVENT.

### Oedema detection: performance of expert readers and ML models 1 and 2

Table 1 shows the distribution of BMO negative (Berlin score = 0) and positive (Berlin score = 1/2/3) assessment according to R1 and R2 over the three reading sessions in MEASURE 1 and PREVENT per VU.

**Table 1.** Number of VUs in MEASURE 1 and PREVENT with BMO negative (Berlin score = 0) and positive (Berlin score = 1/2/3) scoring according to R1 and R2 over three reading sessions

		Reader 1				Reader 2				Both Readers
		Session 1	Session 2	Session 3	Consensus	Session 1	Session 2	Session 3	Consensus	Super-consensus
MEASURE 1	BMO negative (Berlin score = 0)	6565	8990	9670	12 062	6170	8770	9365	11 533	11 418
	BMO positive (Berlin score = 1/2/3)	398	331	285	270	733	702	652	624	202
PREVENT	BMO negative (Berlin score = 0)	23 812	34 514	44 750	43 711	24 027	35 108	45 443	44 564	43 135
	BMO positive (Berlin score = 1/2/3)	1072	1696	1490	996	833	1088	802	557	337

Note that Reader 1, Reader 2, and Both Readers are unique and different for MEASURE 1 and PREVENT.

BMO: bone marrow oedema.

Table 2 shows Readers 1 and 2 intra- and inter-reader agreement and accuracy (averaged across the three reading sessions), specificity and sensitivity in MEASURE 1 and PREVENT. Intra-reader accuracy ranged from 78.8% to 81.7% (with kappa ranging from 0.55 to 0.62), while inter-reader accuracy ranged from 74.2% to 76.8% (with kappa ranging from 0.46 to 0.47).

**Table 2.** Readers 1 and 2 intra- and inter-reader agreement, accuracy (averaged across the three reading sessions), specificity and sensitivity in MEASURE 1 and PREVENT

		Intra-reader		Inter-reader
		Reader 1	Reader 2	
MEASURE 1	Kappa	0.55	0.62	0.47
	Balanced accuracy	78.8%	81.7%	76.8%
	Specificity	0.98	0.97	0.97
	Sensitivity	0.59	0.67	0.57
PREVENT	Kappa	0.60	0.55	0.46
	Balanced accuracy	80.5%	78.8%	74.2%
	Specificity	0.98	0.99	0.98
	Sensitivity	0.63	0.59	0.50

The intra-reader results represent an average of a specific reader’s grading against themselves at different read sessions, e.g. average of Reader (R) 1 Session (S) 1 *vs* R1S2 + R1S2 *vs* R1S3 + R1S2 *vs* R1S3. The inter-reader results are similar to the intra-reader averaged performance but include all the combinations of Reader 1 and Reader 2 at different read sessions, i.e. R1S1 *vs* R2S1 + R1S2 *vs* R2S2 + R1S3 *vs* R2S3. As such, when comparing the separate readings, one set of reading was set as the reference to be compared against.

#### Model 1 performance

Model 1 performance (Table 3) showed a super-consensus AUC of 0.94, an accuracy of 75.8% and a kappa of 0.50, which are equivalent to the inter-reader accuracy of 76.8% and inter-reader kappa of 0.47. Similarly, looking at each reader specifically, Model 1 showed a R1 consensus AUC of 0.92, accuracy of 76.4% and kappa of 0.41, which are comparable to R1’s intra-reader accuracy of 78.8% and kappa of 0.55; Model 1 showed an R2 consensus AUC of 0.83, accuracy of 69.3% and kappa of 0.42, which are lower than R2’s intra-reader accuracy of 81.7% and kappa of 0.62. The specificity and sensitivity values of the Model 1 super-consensus prediction were similar to the inter-reader (R2 *vs* R1) values: 0.99 *vs* 0.97 and 0.52 *vs* 0.57, respectively.

**Table 3.** Performance of machine learning Model 1 in MEASURE 1 and PREVENT, using expert readers’ scoring as the external standard

		Reader 1				Reader 2				Both Readers
		Session 1	Session 2	Session 3	Consensus	Session 1	Session 2	Session 3	Consensus	Super-consensus
MEASURE 1	Kappa	0.41	0.44	0.34	0.41	0.36	0.36	0.36	0.42	0.50
	Balanced accuracy (%)	67.6	71.3	69.0	76.4	67.7	66.8	67.7	69.3	75.8
	AUC	0.84	0.89	0.88	0.92	0.79	0.79	0.80	0.83	0.94
	Specificity	0.97	0.98	0.98	0.98	0.94	0.96	0.96	0.98	0.99
	Sensitivity	0.37	0.44	0.40	0.55	0.42	0.37	0.39	0.41	0.52
PREVENT	Kappa	0.40	0.36	0.36	0.41	0.44	0.42	0.42	0.50	0.52
	Balanced accuracy (%)	70.1	68.1	68.6	69.9	69.7	70.5	70.0	73.9	74.6
	AUC	0.88	0.83	0.89	0.92	0.89	0.89	0.91	0.95	0.97
	Specificity	0.97	0.97	0.98	0.99	0.99	0.98	0.99	0.99	0.99
	Sensitivity	0.43	0.39	0.39	0.41	0.41	0.43	0.41	0.48	0.50

Kappa provides a measure of agreement beyond chance, with values ranging from −1 to 1, where 0 indicates agreement equivalent to chance and higher positive values indicate stronger agreement. Balanced accuracy is the average per-class accuracy which is equal to (Sensitivity + Specificity)/2. AUC is the area under receiver operating characteristic curve. Specificity = Number of True Negatives/(Number of True Negatives + Number of False Positives). Sensitivity = Number of True Positives/(Number of True Positives + Number of False Negatives). For comparison, compare reader-specific performance against intra-reader values in Table 3. The consensus score is vertebral unit (VU) specific. For Reader 1’s consensus results, a VU can only be marked as positive or negative if Reader 1 Session 1 (R1S1) agrees with Reader 1 Session 2 (R1S2) and with Reader 1 Session 3 (R1S3). In essence, the VU label is determined by VU=R1S1 ^ R1S2 ^ R1S3, where ‘^’ represents a logical AND operation, but only cases where all read sessions are in agreement are used. If there is any disagreement, the VU is excluded. The super-consensus, on the other hand, applies to cases where both readers (Reader 1 and Reader 2) agree in all read sessions.

In PREVENT, Model 1 (trained only on MEASURE 1) showed a super-consensus AUC of 0.97, an accuracy of 74.6% and a kappa of 0.52, which are equivalent to the inter-reader accuracy of 74.2% and inter-reader kappa of 0.46. Similarly, looking at each reader specifically, Model 1 showed an R1 consensus AUC of 0.92, accuracy of 69.9% and kappa of 0.41, which are lower than R1’s intra-reader accuracy of 80.5% and kappa of 0.60; Model 1 showed a R2 consensus AUC of 0.95, accuracy of 73.9% and kappa of 0.50, which are closer to R2’s intra-reader accuracy of 78.8% and kappa of 0.55, compared with the differences observed for R1. The specificity and sensitivity values of the Model 1 super-consensus prediction were similar to the inter-reader (R2 *vs* R1) values: 0.99 *vs* 0.98 and 0.52 *vs* 0.50, respectively.

#### Model 2 performance

Model 2 performance compared with the expert readers’ scores can be seen in Table 4.

**Table 4.** Performance of machine learning Model 2 in MEASURE 1 and PREVENT, using expert readers’ scoring as the external standard

		Reader 1				Reader 2				Both Readers
		Session 1	Session 2	Session 3	Consensus	Session 1	Session 2	Session 3	Consensus	Super-consensus
MEASURE 1	Kappa	0.32	0.31	0.24	0.30	0.31	0.30	0.27	0.30	0.31
	Balanced accuracy (%)	68.1	66. 9	66.5	68.7	68.1	69.1	64.8	65.8	70.5
	AUC	0.83	0.85	0.79	0.84	0.78	0.80	0.77	0.82	0.88
	Specificity	0.95	0.97	0.96	0.97	0.89	0.91	0.94	0.96	0.98
	Sensitivity	0.41	0.37	0.37	0.40	0.47	0.47	0.36	0.36	0.43
PREVENT	Kappa	0.24	0.24	0.23	0.23	0.34	0.31	0.32	0.34	0.27
	Balanced accuracy (%)	63.7	64.0	64.0	66.7	67.7	66.2	69.2	70.3	70.0
	AUC	0.79	0.77	0.80	0.83	0.85	0.84	0.86	0.91	0.91
	Specificity	0.96	0.95	0.96	0.97	0.98	0.98	0.98	0.99	0.99
	Sensitivity	0.32	0.33	0.32	0.37	0.38	0.35	0.50	0.42	0.41

Kappa provides a measure of agreement beyond chance, with values ranging from −1 to 1, where 0 indicates agreement equivalent to chance and higher positive values indicate stronger agreement. Balanced accuracy is the average per-class accuracy which is equal to (Sensitivity + Specificity)/2. AUC is the area under receiver operating characteristic curve. Specificity = Number of True Negatives/(Number of True Negatives + Number of False Positives). Sensitivity = Number of True Positives/(Number of True Positives + Number of False Negatives). For comparison, compare reader specific performance against intra-reader values in Table 3. The consensus score is vertebral unit (VU) specific. For Reader 1’s consensus results, a VU can only be marked as positive or negative if Reader 1 Session 1 (R1S1) agrees with Reader 1 Session 2 (R1S2) and with Reader 1 Session 3 (R1S3). In essence, the VU label is determined by VU=R1S1 ^ R1S2 ^ R1S3, where ‘^’ represents a logical AND operation, but only cases where all read sessions are in agreement are used. If there is any disagreement, the VU is excluded. The super-consensus, on the other hand, applies to cases where both readers (Reader 1 and Reader 2) agree in all read sessions.

In MEASURE 1, Model 2 showed a super-consensus AUC of 0.88, an accuracy of 70.5% and a kappa of 0.31 compared with Model 1’s AUC of 0.94, accuracy of 75.8% and kappa of 0.50. Similarly, in PREVENT, Model 2 (also trained only on MEASURE 1) reported a super-consensus AUC of 0.91, an accuracy of 70.0% and a kappa of 0.27, which were lower than Model 1’s AUC of 0.97, accuracy of 74.6% and kappa of 0.52. The same performance drop can be seen in the sensitivity values when comparing Model 1 *vs* Model 2 in MEASURE 1: specificity of 0.99 *vs* 0.98 and sensitivity of 0.52 *vs* 0.43; and in PREVENT: specificity of 0.99 *vs* 0.99 and sensitivity of 0.50 *vs* 0.41, respectively.

## Discussion

In this study, we developed a fully automated ML system to classify the presence or absence of BMO lesions in whole-spine sagittal MRI scans. The automated assessment of MRIs in axSpA patients using this ML software was found to be comparable to assessments made by expert readers. We tested two models: Model 1, without manual segmentation, and Model 2, incorporating an intermediate manual segmentation step. Model 1, which did not require manual input, outperformed Model 2.

MRI is widely accepted for evaluating disease activity in axSpA, and quantifying MRI changes has become crucial for demonstrating the efficacy of advanced targeted therapies. However, large-scale MRI reading campaigns are time-consuming, costly and subject to inter- and intra-reader variability. In the r-axSpA MEASURE 1 study, we employed pre-existing ML software to detect and label spinal vertebrae in MRI scans, comprising 23 vertebrae per scan. We successfully trained an ML algorithm using human labels based on a previously developed software called SpineNet [13] and its updated variant, SpineNetV2 [13, 26]. The ML algorithm was able to replicate human expert assessment with performance comparable to intra- and inter-reader agreement. Model 1 (without manual segmentation) outperformed Model 2 (with manual segmentation), with an AUC of 0.94 (*vs* 0.88), accuracy of 75.8% (*vs* 70.5%) and kappa of 0.50 (*vs* 0.31), using super-consensus assessment as the external reference. This was similar to the expert inter-reader accuracy of 76.8% and kappa of 0.47 in the MEASURE 1 dataset. In a nr-axSpA dataset (PREVENT), Model 1 achieved an AUC of 0.97 (*vs* 0.91 for Model 2), accuracy of 74.6% (*vs* 70% for Model 2) and kappa of 0.52 (*vs* 0.27 for Model 2), also comparable to expert inter-reader accuracy.

Model 2’s lower performance compared with Model 1 likely stems from multiple factors inherent to segmentation-based approaches. Manual segmentation introduces variability due to differences in expert annotation, which may affect consistency in model training. Additionally, the dataset size might not be sufficient for optimal segmentation learning, as segmentation-based models typically require larger annotated datasets to refine boundary detection and lesion classification. Furthermore, lesion segmentation itself presents challenges: subtle intensity variations, overlapping structures and anatomical complexity can hinder precise delineation, impacting downstream classification performance. In contrast, Model 1 benefits from direct feature extraction across the full image without segmentation constraints. Future improvements to segmentation-based models may include advanced annotation techniques, self-supervised learning approaches or larger, high-quality training datasets to enhance accuracy.

Previous work has focused on ML systems detecting inflammation in the sacroiliac joints (SIJs), with only one study, by Lin *et al.* [22] addressing the spine. Published ML models for the SIJs can be classified into two categories: (i) semi-automatic pipelines requiring human involvement, such as those by Kucybała *et al.* [14], Zarco *et al.* [15] and Garrido-González *et al.* [16] and (ii) fully automated pipelines as seen in the studies by Ożga *et al.* [17], Rzecki *et al.* [18], Bressem *et al.* [19], Lee *et al.* [20] and Nicolaes *et al.* [21]. Comparing our results with the only study performed at the spinal level, by Lin *et al.* [22], our best-performing model, with an AUC of 0.94, exceeded their reported AUC of 0.88. Our approach differs from that of Lin *et al.* in two key aspects: (i) Model 1 is fully automated, requiring no manual region of interest (ROI) annotation, and (ii) our classification pipeline uses both T1-weighted and STIR sequences, whereas Lin *et al.* relied solely on STIR.

There are limitations to our study. First, it was conducted using clinical trial data, which may not be fully representative of real-world scenarios due to controlled settings and selective participant inclusion. This raises concerns about the generalizability of our findings to routine clinical imaging. Clinical trials often use standardized protocols and specific scanner types, which may differ from routine clinical practice. However, this study employed an MRI protocol that aligns with current clinical practice at most sites, allowing the use of any available scanner brand; this approach enhances the generalizability of the study. Additionally, the participant population in clinical trials may not reflect the diversity seen in real-world settings. Further research is needed to validate our findings using real-world observational datasets, ensuring the model’s performance remains robust across various clinical environments.

Second, we developed a binary ML classification system to identify the presence or absence of spinal BMO, rather than a model that attempts to quantify the extent or intensity of inflammatory lesions. This decision was driven by our stepwise modelling approach, in which the initial aim was to develop a robust and generalizable system for detecting any BMO. We recognize that dichotomizing the Berlin score simplifies the underlying spectrum of inflammatory activity and may mask clinically relevant gradations in severity. However, modelling the full ordinal range of BMO requires more complex architectures and more balanced datasets, with a greater proportion of non-zero BMO scores than were available in the current study. Developing models that can accurately capture the extent and intensity of inflammation is a key next step, and one that we are actively pursuing. Such models have the potential to enhance the clinical and research utility of ML tools by enabling more nuanced monitoring of disease activity and treatment response.

Third, we did not assess the ability to detect changes over time. Future work should explore this using longitudinal datasets. To better evaluate disease progression and treatment response, an ML-based scoring system could be developed to quantify changes in imaging findings over time. This system would need to establish clinically meaningful thresholds for lesion evolution, ensuring that it reflects true biological changes rather than measurement variability. Importantly, its responsiveness to disease progression and therapeutic effects must be rigorously validated. Assessing the system’s sensitivity to change would be essential to determine whether the scoring system can differentiate between stable disease, improvement and progression, ensuring its utility in both research—namely, clinical trials—and clinical applications.

Fourth, the relatively low kappa values observed in this study should be interpreted with caution, as kappa can be sensitive to class imbalance and may not fully reflect the reliability of the ML algorithm. A substantial proportion of VUs had zero scores, skewing the dataset and influencing the calculation of kappa. Despite this, the model’s kappa values were comparable to those observed in human inter-reader variability exercises, indicating that its agreement levels align with expert assessments. While kappa alone does not provide a complete picture of model performance, the additional metrics presented—namely, sensitivity, specificity and balanced accuracy—offer complementary insights into the robustness and clinical applicability of the ML system.

Finally, it is important to emphasize that this study exclusively focused on patients diagnosed with axSpA. The ML algorithm was specifically designed to detect BMO within this context, rather than for differential diagnosis, distinguishing axSpA-related BMO from other causes—such as biomechanical stress or degenerative changes—or capturing variations in disease presentation.

The integration of both T1-weighted and STIR imaging data strengthens the study’s methodological rigor. By incorporating both sequences into the ML evaluation, the approach ensures a more comprehensive and contextual imaging assessment, reinforcing the robustness and reliability of our results. Another advantage of our ML models is that the threshold for BMO detection can be adjusted depending on clinical context. While the primary objective of this study was to replicate expert assessment, different applications may require alternative optimization strategies. For example, in detecting low levels of inflammation or monitoring treatment response, a higher sensitivity may be desirable to minimize false negatives. In such cases, the model threshold can be lowered to increase sensitivity, accepting a corresponding reduction in specificity. Conversely, in contexts where false positives carry greater clinical or therapeutic consequences—such as the risk of overdiagnosis in a low pre-test probability setting—maintaining a higher threshold to prioritize specificity may be preferable. This flexibility allows the model to be tailored to diverse clinical scenarios. However, a formal trade-off analysis—evaluating sensitivity, specificity and predictive value across different threshold settings—was beyond the scope of the current study and should be addressed in future work. Such analyses will be particularly important when integrating ML tools into clinical pathways, where decision-making often depends on the relative risk tolerance of a given application. Beyond threshold tuning, additional strategies may improve model sensitivity. These include more sophisticated data augmentation techniques to address class imbalance, incorporation of additional imaging sequences (e.g. multiplanar STIR and T1-weighted sequences) and inclusion of relevant clinical metadata. We are actively exploring these avenues to enhance model performance, particularly in detecting lower-grade or borderline inflammatory lesions.

In conclusion, our study suggests that ML-based software offers significant potential for automating MRI assessment. Automated assessment could reduce the time and effort required for manually assessing MR images, enhance accessibility, minimize variability and support large-scale studies. Future studies should include comparisons with quantitative grading systems and an assessment of sensitivity to change.

## Supplementary material

Supplementary material is available at *Rheumatology* online.

## Supplementary Material

keaf323_Supplementary_Data

## Data Availability

The datasets (MEASURE 1, PREVENT) used for training and testing were obtained from completed, anonymized clinical trials. Because both the clinical trial data and the images are anonymized, these are no longer personal data and so no further EC/IRB (Ethics Committees/Institutional Review Board) approvals are required. Novartis is committed to sharing with qualified external researchers’ access to patient-level data and supporting clinical documents from eligible studies. These requests are reviewed and approved based on scientific merit. All data provided are anonymized to respect the privacy of patients who have participated in the trial in line with applicable laws and regulations. This trial data availability is according to the criteria and process described on www.clinicalstudydatarequest.com.
