# Supplementary material for: Automated detection of spinal bone marrow oedema in axial spondyloarthritis: training and validation using two large phase 3 trial datasets
Source: Rheumatology (Oxford). 2025 Jun 9;64(10):5446–54. doi: 10.1093/rheumatology/keaf323 (PMC12494195; doi:10.1093/rheumatology/keaf323)
Supplement: keaf323_Supplementary_Data [file keaf323_supplementary_data.docx]

**Automated Detection of Spinal Bone Marrow Oedema in Axial Spondyloarthritis:**

**Training and Validation Using Two Large Phase 3 Trial Datasets**

**Supplementary Data S1. Supplementary Methods**

**MEASURE 1 patient population:** Briefly, patients with a clinical diagnosis of radiographic axSpA (r-axSpA, or ankylosing spondylitis [AS]) aged ≥18 years were included if they met the modified New York criteria for AS. Eligible patients had a score of 4 or higher on the Bath Ankylosing Spondylitis Disease Activity Index (BASDAI; 0-10) and a score for spinal pain of 4 cm or more on a 10-cm visual-analogue scale (VAS), despite treatment with the maximum doses of NSAIDs that were associated with an acceptable side-effects profile. Patients previously treated with not more than one TNFi agent could participate if they had an inadequate response to an approved dose for 3 months or more or had unacceptable side effects with at least one dose (inadequate response to anti-TNF agents). Patients could continue to receive the following medications at a stable dose: sulfasalazine (≤3 g per day), methotrexate (≤25 mg per week), prednisone or equivalent (≤10 mg per day), and NSAIDs.

**PREVENT patient population:** Briefly, patients with a clinical diagnosis of non-radiographic axSpA (nr-axSpA) aged ≥18 years were included if they met the ASAS classification criteria for axSpA, along with objective signs of inflammation—either SIJ inflammation on MRI (confirmed by central reading) or elevated high-sensitivity C-reactive protein (hsCRP) levels above the upper limit of normal as defined by the central laboratory. Active disease was required at baseline, defined by a total BASDAI score of ≥4 cm (on a 0–10 cm scale), spinal pain (BASDAI question 2) of ≥4 cm, and total back pain (VAS) of ≥40 mm (on a 0–100 mm scale), despite treatment with the maximum doses of NSAIDs that were associated with an acceptable side-effects profile. Patients previously treated with a TNFi (limited to one prior agent) could be enrolled if they had experienced an inadequate response or intolerance. Those included in the study were permitted to continue stable doses of sulfasalazine (≤3 g/day), methotrexate (≤25 mg/week), corticosteroids (≤10 mg/day of prednisone or its equivalent), and NSAIDs.

**MRI spine protocol in MEASURE and PREVENT:** Images were acquired locally using 1.5T or 3.0T scanners (maintaining consistency for each subject throughout the trial) from any available brand (GE, Siemens, Philips, or Canon Medical [formerly Toshiba]) at participating sites. Two spinal segments were scanned without contrast, ensuring complete coverage with slight overlap: cervical & upper thoracic (C2–T10; slice thickness 3 mm) and lower thoracic & lumbar (T8–S1; slice thickness 4 mm). Sagittal T1 sequences without fat suppression (TR 400–500 ms [or optimal]; TE 21 ms [or optimal]) and sagittal STIR sequences (TR 4500 ms [or optimal]; TE 29 ms [or optimal], extended to 37 ms for 3T where applicable; TI 150 ms [or 190 ms for 3T]) were obtained. MRI images were transferred as anonymised electronic files to the central imaging lab following acquisition, and underwent quality control.

**Supplementary Figures:**

**Supplementary Figure S1. Vertebrae detection and labelling pipeline.** The detection and labelling pipeline takes sagittal spinal MRIs (with various fields of view, such as whole spine, lumbar, cervicothoracic, etc.) as input (left panel), and outputs the position of each vertebral body along with their predicted level labels from C3 to S1 (right panel).

**
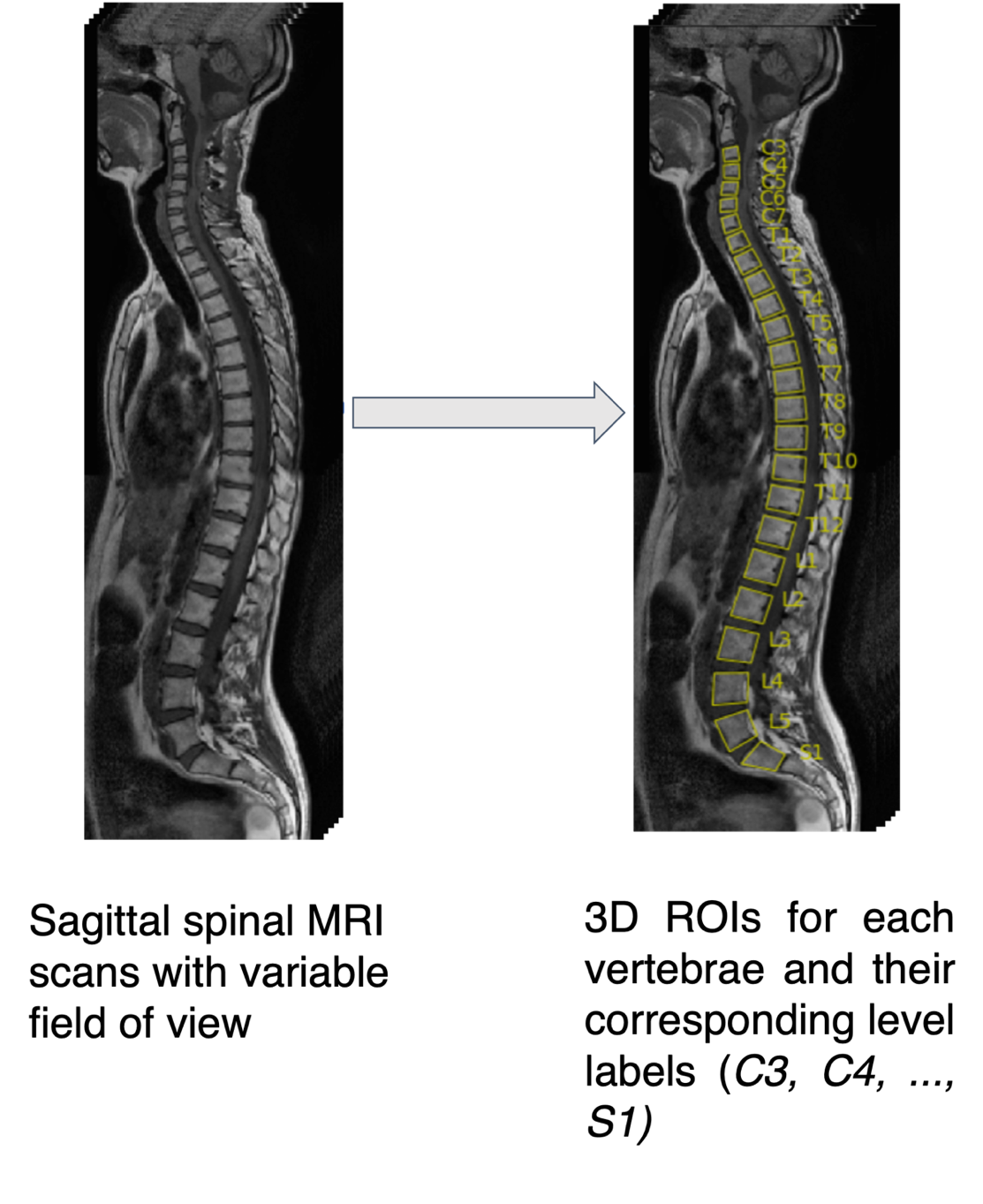
**

**Supplementary Figure S2. Detection and labeling for a given patient in MEASURE 1 across multiple time points.** Shown below the midsagittal slices of the scans are the extracted vertebral bodies´ midsagittal slices.

**
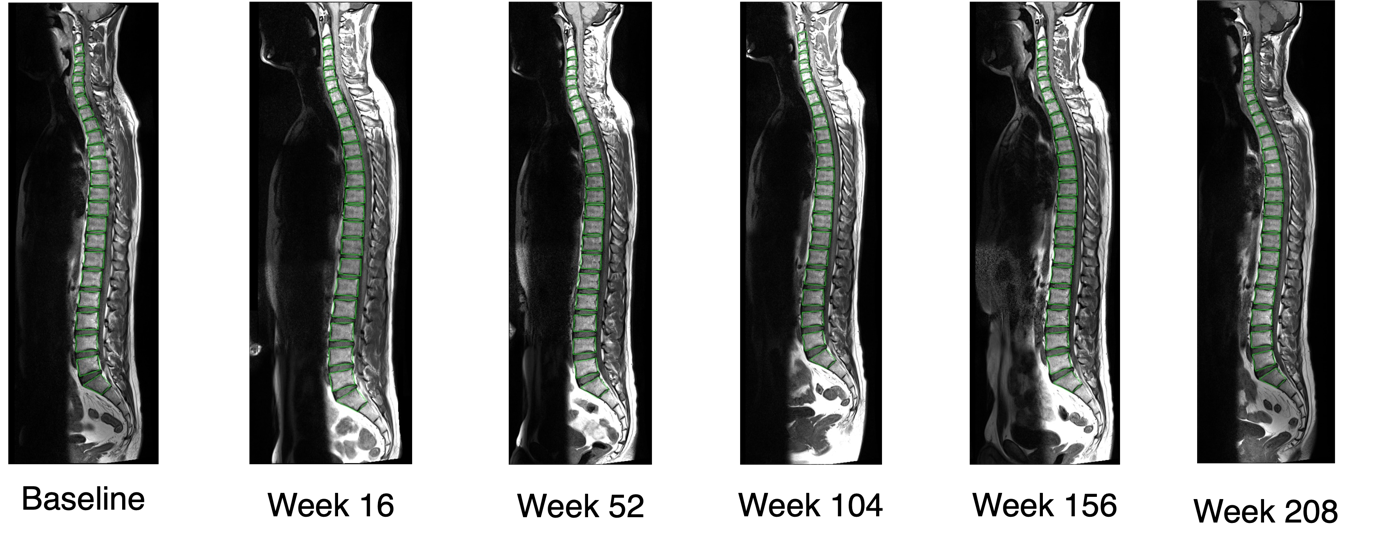
**
